# Promotion of beta cell proliferation through DYRK kinase inhibition using the marine natural product breitfussin C

**DOI:** 10.1038/s41598-025-85178-w

**Published:** 2025-01-08

**Authors:** Sara Ullsten, Kine Østnes Hansen, Guillaume Axel Petit, Espen Holst Hansen, Jeanette Hammer Andersen

**Affiliations:** MARBIO, UiT – The Arctic University of Norway, Breivika, 9037 Tromsø, Norway

**Keywords:** Diabetes, Beta cells, Proliferation, Kinase inhibition, DYRK, Cytokines, Drug discovery and development, Diabetes

## Abstract

Pro-inflammatory cytokines, like interleukin-1 beta and interferon gamma, are known to activate signalling pathways causing pancreatic beta cell death and dysfunction, contributing to the onset of diabetes. Targeting cytokine signalling pathways offers a potential strategy to slow or even halt disease progression, reducing reliance on exogenous insulin and improving glucose regulation. This study explores the protective and proliferative effects of breitfussin C (BfC), a natural compound isolated from the Arctic marine hydrozoan *Thuiaria breitfussi*, on pancreatic beta cells exposed to pro-inflammatory cytokines. Using the beta cell line RIN-M5F, we assessed the protective effects of BfC through a MTS assay for cell viability, caspase 3/7 activity for apoptosis, and EdU incorporation and cell cycle distribution for proliferation. Additionally, we investigated BfC’s inhibitory effects on the DYRK family of kinases using kinase activity and binding assays, western blotting, and docking simulations. Our findings reveal that BfC treatment effectively increases beta cell proliferation and counteracts cytokine-induced decrease in proliferation. The proliferative effect is associated with inhibition of DYRK kinases and a subsequent decrease in the cell cycle inhibitor p27^KIP^. These results suggest that BfC mediates beta cell-protective effect by promoting proliferation through DYRK inhibition, highlighting its potential as a molecular starting point for the development of a therapeutic agent against diabetes.

**Supplementary Information:**

The online version contains supplementary material available at 10.1038/s41598-025-85178-w.

## Introduction

Beta cell numbers are significantly reduced in patients with diabetes. In type 2 diabetes, patients lose approximately half of their functionally active endocrine mass, and in type 1 diabetes, it is commonly stated that symptoms occur when about 90% of the beta cell mass is destroyed^1,2^. To minimize the loss of endocrine mass during disease progression, extensive efforts to identify new strategies to either halt the immune attack or expand the existing cell mass are ongoing. Increasing the number of insulin-producing beta cells represents a promising therapeutic strategy that would improve the clinical outcome in both type 1 and type 2 diabetes patients by enhancing insulin secretion, improving glucose homeostasis and reducing the risk of secondary complications. Currently, there are no available treatments for induction of beta cell proliferation, however, several compounds are under investigation including serpin B1^3^, γ-aminobutyric acid (GABA)^4^, osteoprotegerin and denosumab^5,6^, parathyroid hormone-related protein^7^, GLP1 receptor analogues^8^ and the TGF-β superfamily^9^. Unfortunately, most investigated proliferative agents have major drawbacks, such as low impact on beta cell proliferation and off-target proliferation^10^. These constraints present significant challenges in the progression of these agents toward becoming viable therapeutic pharmaceuticals.

Kinase deregulation has been observed in many important diseases, including autoimmune-, inflammatory-, and cardiovascular diseases, central nervous system disorders and viral infections, making them important targets for drug development^11^. In the context of diabetes, it has been suggested that Tyrosine Kinase Inhibitors (TKIs) interfere with internal immune/cytokine signalling^12^. Consistent with this, several reports have shown that diabetic patients receiving cancer treatment with TKIs experience improvement in glycaemic control^12^.

The possibility of targeting cell cycle regulation through kinase inhibition to regulate beta cell proliferation has recently gained widespread attention. Members of the dual-specificity tyrosine-regulated kinase (DYRK) family have been described as a particularly promising targets due to their ability to induce strong and partial cell type-specific increase in beta cell proliferation^13–15^. Many agents inhibiting DYRK kinases are natural products and screening for DYRK inhibitors from natural resources has resulted in the identification of the compounds harmine, 5IT, aristolactam BIII, EGCG, acrifoline, leucettines, and meridianins^10,16–19^.

**Fig. 1:**
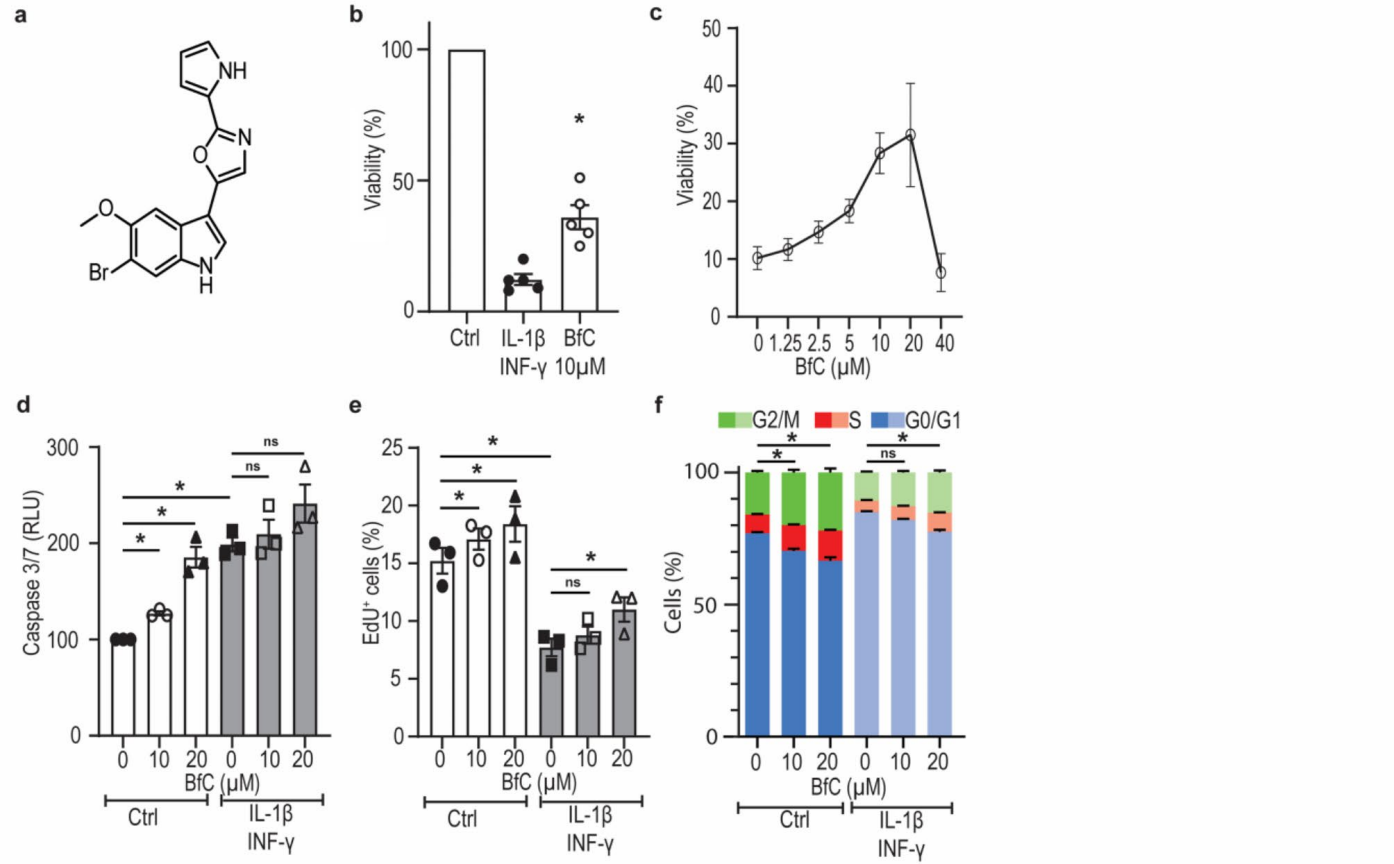
Breitfussin C (BfC) protects RIN-M5F cells from cytokine-induced cell death by inducing proliferation. (**A**): BfC molecular structure. (**B**): Cell viability after cytokine treatment (20 ng/ml IL-1β and 20 ng/ml IFNγ), with and without 10 µM BfC. Treatment with 10 µM BfC significantly increased cell viability compared to cytokine treatment alone. Data represent mean ± SEM of five individual experiments. * *p* < 0.05 compared with control, two-tailed Student t test. (**C**): Dose-response curve of cell viability in RIN-M5F cells exposed to cytokines and BfC. Data represent mean ± SEM of three individual experiments. (**D**): Relative Caspase 3/7 activity in RIN-M5F cells following treatment with cytokines, with or without co-treatment of 10 µM and 20 µM BfC. Data represent mean ± SEM of three individual experiments. *p < 0.05 compared to control, one-way ANOVA with Bonferroni’s post hoc test. (**E**): EdU incorporation in untreated and cytokine-treated cells, with and without co-treatment of 10 µM and 20 µM BfC. Data represent mean ± SEM of three individual experiments. *p < 0.05 compared to control, RM one-way ANOVA with Bonferroni’s post hoc test. (**F**): Cell cycle distribution following treatment with 20 ng/ml IL-1β and 20 ng/ml IFNγ, with or without co-treatment of 10 µM and 20 µM BfC. In cells without cytokines, 10 µM and 20 µM BfC significantly decreased cells in G0/G1 phase, significantly increased cells in S and G2/M phase. In cells treated with cytokines, only 20 µM BfC decreased the number of cells in G0/G1 and significantly increased the cells in S and G2/M phase. Data represents mean ± SEM of three individual experiments, *p* < 0.05 were considered statistically significant analyzed by two-way ANOVA with Dunnett’s post hoc test. ns: not significant.

The breitfussins, a family of halogenated compounds with a novel indole-oxazole-pyrrole molecular scaffold, were previously isolated by our research group from the Arctic, marine hydrozoan *Thuiaria breitfussi*. The family consists of eight compounds, breitfussin A-H (BfA-H)^20,21^. Based on previous bioactivity characterisation of BfA-H, selected variants exhibited kinase inhibition properties, out of which BfC (Fig. 1a) was the superior variant in terms of kinase inhibition potency and selectivity^20^. However, previous studies have not pinpointed specific pathophysiological disorders where the heterocyclic scaffold of the breitfussins can be utilised for drug development. In this study, we conduct further investigations into the bioactivity of breitfussin C (BfC), elucidating its capacity for DYRK inhibition and induction of beta cell proliferation, highlighting the breitfussin scaffold as a viable starting point for further investigation into becoming a treatment option for type 1 and type 2 diabetes.

## Materials and methods

### Compound synthesis

BfC was synthetically produced as previously described in Hansen et al.^20^.

### Cell culture and cytokine-induced cell death

Rat beta cells RIN-M5F (purchased from ATCC, Manassas, VA, USA, product code ATCC-CRL-11605, passage number below 40 for all experiments presented in this manuscript) were cultured in RPMI-1640 (cat# R5886, Sigma-Aldrich, Saint Louis, MO, USA) supplemented with 10% FBS (Biowest, Nauillé, France), 2 mM L-glutamine (Biowest), 1 mM sodium pyruvate (Merck, Darmstadt, Germany) and 1x Penicillin-Streptomycin (Sigma-Aldrich) according to manufacturer’s instructions. Cells were seeded 3 days before the start of each experiment to allow full attachment. In experiments analysing the effects of cytokines, cells were treated with 20 ng/ml human IL-1β and 20 ng/ml rat IFNγ (Peprotech, Rocky Hill, NJ, USA) for a given time as indicated. Mycoplasma was tested as negative using MycoAlert^®^ Mycoplasma Kit (Lonza, Basel, Switzerland).

### Analysis of cell viability

RIN-M5F cells were cultured in 96-well plates, 2 × 10^4^ cells/well. BfC at a concentration of 10 µM was added to the cells. In dose-response experiments, BfC was two-fold serially diluted between 40 µM and 1.25 µM and added to the cells. Control wells with RPMI-1640 alone or supplemented with DMSO (Sigma-Aldrich, final concentration 10%) were used to estimate absorbance at 100% or 0% viability, respectively. 24 h after addition of substances, cytokines (IL1-β and IFNγ) were added to the experiment. The cells were thereafter incubated for an additional 72 h, and cell viability was measured by CellTiter 96^®^AQueous One Solution Assay (Promega, Madison, WI, USA) according to the manufacturer’s instructions. Absorbance at 490 nm was measured using a Spark^®^ multimode microplate reader (Tecan, Männedorf, Switzerland). Each experiment was performed in triplicates.

### Analysis of caspase 3/7 activity

RIN-M5F cells were cultured in 96-well plates, 2 × 10^4^ cells/well. Caspase 3/7 activity was analysed in cells treated for 48 h with 10 or 20 µM of BfC, with or without cytokines. After 24 h of BfC treatment, cytokines were added, and the cells were co-stimulated for an additional period of 24 h. Caspase 3/7 activity was measured by Caspase-Glo (Promega) according to the manufacturer’s instructions. Each experiment was performed in triplicates.

### EdU incorporation

RIN-M5F cells were seeded into 6 well plates, 6 × 10^5^ cells per well. Staining with 5-ethynyl 2′-deoxyuridine (EdU) was performed on cells treated with 10 or 20 µM of BfC, with or without cytokines. After 24 h of BfC treatment, cytokines were added, and the cells were stimulated for a total of 48 h. Subsequently, the cells were stained with 10 µM Click-iT EdU (Invitrogen, Waltham, MA, USA) for 2 h, and analysed by flow cytometry (CytoFLEX, Beckman Coulter, Brea, CA, USA) according to the manufacturer’s instructions. EdU incorporation was analysed using the CytExpert software.

### Cell cycle

RIN-M5F cells were seeded in 6-well plates, 6 × 10^5^ cells/well. Cell cycle distribution was analysed in cells treated for 48 h with 10 or 20 µM of BfC, with or without cytokines. After 24 h of BfC treatment, cytokines were added, and the cells were co-stimulated for an additional period of 24 h. After stimulation, the cells were washed with phosphate buffered saline (PBS) and trypsinated for 5 min at 37 °C, and fixed in ice-cold ethanol (70%) at 4 °C until analysis. The cells were stained by FxCycle™ PI/RNase Staining Solution kit (Invitrogen) according to manufacturer’s instructions and analysed by flow cytometry (CytoFLEX, Beckman Coulter). The percentages of cells in G1, S1 and G2/M was determined using the CytExpert software.

### Kinase activity assay

The IC_50_ values of BfC against the DYRK1A, DYRK2 and DYRK3 kinases were measured using ^33^P-ATP radioactive filter binding assays^22^ at the International Centre for Kinase Profiling. Each kinase was assayed using a duplicate 10-point concentration curve.

### Lantha assay

Kinase binding activity was performed as described in^23^. Briefly, a 12-points dilution series of the compounds was prepared in DMSO and further diluted 1:33 in assay buffer A (50 mM HEPES (Sigma-Aldrich) pH 7.5, 10 mM MgCl_2_ (Sigma-Aldrich) 1 mM EGTA (Sigma-Aldrich) and 0.01% BRIJ-35 (ThermoFisher Scientific, Waltham, MA, USA). The GST tagged kinases, antibodies and tracer were purchased from ThermoFisher Scientific. DYRK1A and DYRK1B were each mixed with rabbit anti GST Europium tagged antibody in assay buffer A at concentrations of 15 nM kinase and 6 nM antibodies. Tracer 236 was diluted to 90 nM (DYRK1A) or 30 nM (DYRK1B) in assay buffer A. Finally, the kinase-antibody solution, compound solution and tracer solution were mixed together 1:1:1 (5µL each) in white, non-binding, round bottom 384 well microplates (Corning Inc, Kennebunk, ME, USA) mixed at 1000 rpm for 1 min and incubated in the dark at RT in humid atmosphere for 1 h. Fluorescence measurements were taken using a EnVision 2104 Multilabel reader (PerkinElmer, Shelton, CT, USA), excitation wavelength was set to 340 nm and emission was read at both 615 nm (8.5 nm bandwidth) and 665 nm (7.5 nm bandwidth) over 200ms with a 100ms delay between excitation and emission measurements. Emission ratio signals (EM) were calculated as the ratio between the 665 nm signal and the 615 nm signal. Mean EM values of technical triplicate were plotted against compound concentration and used to calculate an IC_50_ first using the default “three parameter log (inhibition) vs. response” function from Prism 10.1. for Windows (GraphPad Software, Boston, Massachusetts USA). Because BfC autofluorescence was interfering with the fluorescent readings, the fitting formula was also adjusted as described in^24^ and the parameter “bot” was fixed to values obtained from control compound 5IT to ensure a correct range.

### Docking

BfC was docked into the ATP-binding pocket of DYRK1A using Protein Data Bank (PDB) model 6S17^25^. BfC and control compounds harmine and 5IT were prepared using LigPrep (Schrödinger, Inc., New York, NY, USA, release 2022-2) and the DYRK1A model was prepared (addition of missing hydrogen, fixing different conformers, adding missing residues and energy minimizing the structure) with the Maestro protein preparation wizard (Schrödinger, Inc.)^26^. Docking was performed using Glide extra precision algorithm^27,28^.

### Western blot

RIN-M5F cells were seeded in 6-well plates, 6 × 10^5^ cells/well. After treatment with 10 and 20 µM of BfC for 48 h, the cells were washed with ice-cold PBS and lysed in Pierce^®^ RIPA buffer (Thermo Fischer Scientific, Waltham, MA, USA) supplemented with cOmplete™ Protease Inhibitor Cocktail (Roche, Basel, Switzerland) and PhosSTOP™ (Roche). After centrifugation (16.9 RCF, 15 min at 4 °C) the samples were directly diluted in NuPAGE™ LDS Sample Buffer (4X, Invitrogen) supplemented with NuPAGE™ Sample Reducing Agent (10X, Invitrogen), boiled at 95 °C for 5min and loaded on gel (NuPAGE™ 4–12%, Bis-Tris, Invitrogen). Protein transfer to PVDF membrane was performed by the iBlot™ dry blotting system (Invitrogen) according to manufacturer’s instructions. The membrane was blocked using SuperBlock™ Blocking Buffer (Thermo Fisher Scientific) and subsequently cut in half around 60 kDA, the top half was incubated with primary antibodies for phospho-dyrk1a/b (Tyr321, Tyr273) (1:500, Invitrogen, ref: PA5-64574), while the bottom half was incubated with phospho-p27^kip1^ (Ser10) (1:200, Invitrogen, ref:34-6300) over night at 4^o^C. After washing, the membrane was incubated with Alexa488-conjugated secondary antibody (1:5000, goat anti-rabbit, Invitrogen) for 2 h and imaged with a PharoxFX imager (Bio-Rad, Hercules, CA, USA). Consequently, the bottom half of the gel was washed and re-stained with primary beta-actin antibodies (1:5000, Invitrogen), and then prepared as described for p-p27^kip^, and reimaged. Protein quantification was performed with ImageJ (National Institutes of Health, Bethesda MD, USA), using the gel analysis tool to get the integrated pixel intensity for each band.

### Statistical analysis

Data are expressed as mean ± SEM or SD. All statistical analyses were performed using GraphPad Prism v.7.0 (GraphPad Software, San Diego, CA, USA). Statistical significance was defined as *p* < 0.05 and determined by unpaired Student’s t test, one-way ANOVA with Bonferroni’s post hoc test, RM one-way ANOVA with Bonferroni’s post hoc test or two-way ANOVA with Dunnett’s post hoc test, as appropriate.

## Results

### BfC increases cell viability in cytokine treated cells

Incubation with pro-inflammatory cytokines decreased cell viability to approximately 12% relative to untreated cells. Co-treatment with BfC induced partial protection against the cytokine-induced cell death, with a resultant increase in survival rate to 36% of untreated cells (Fig. 1b, *n* = 5). Dose-response experiments with BfC demonstrated effective protection against cytokine-induced cell death in the range of 10–20 µM, however the protective effect was lost at higher concentrations (Fig. 1c, *n* = 5).

### BfC does not promote cell survival by protection against apoptosis

BfC treatment induced caspase 3/7 activity at 10 and 20µM (127 ± 2% and 186 ± 19% compared to control, respectively, *n* = 3). Cytokines alone approximately doubled caspase 3/7 activity (199%±7, compared to control, *n* = 3). Pretreatment with 10 and 20 µM BfC 24 h before cytokine treatment additionally increased the caspase 3/7 activity to 210 ± 15% and 241 ± 20% compared to control, respectively (Fig. 1d, *n* = 3).

### BfC induces proliferation in RIN-M5F rodent cells

In untreated cells, an EdU + labelling rate of 15.2 ± 1.1% was measured. In cells treated with 10 and 20 µM BfC, EdU + labelling increased to 17.1 ± 0.9% and 18.4 ± 1.5%, which corresponds to an average increase of 12.5% and 21.1%, respectively (*n* = 3). Cytokine treatment decreased the EdU + labelling rate to 7.7 ± 0.8%. Pretreatment with 10 or 20 µM BfC increased the EdU + labelling in cytokine treated cells to 8.8 ± 0.8 and 11.0 ± 1.1%, respectively (Fig. 1e, *n* = 3).

### BfC induces cell cycle shift from G0/G1 to S and G2/M

In cells without cytokines, 10 µM and 20 µM BfC significantly decreased cells in G0/G1 phase and increased cells in S and G2/M phase (Fig. 1f, *n* = 3). Cytokine treatment significantly increased the number of cells in G0/G1 from 76.9 ± 0.6% to 85.0 ± 0.4%. In cells treated with cytokines, only 20 µM BfC significantly decreased the number of cells in G0/G1 and increased the cells in S and G2/M phase (Fig. 1f, *n* = 3). The largest effect of BfC was seen in cells without cytokines, after treatment with 20 µM BfC, where the number of cells in G0/G1 decreased from 76.9 ± 0.6% to 66.6 ± 1.3%.

### BfC calculated interaction with the DYRK ATP binding pocket

**Fig. 2:**
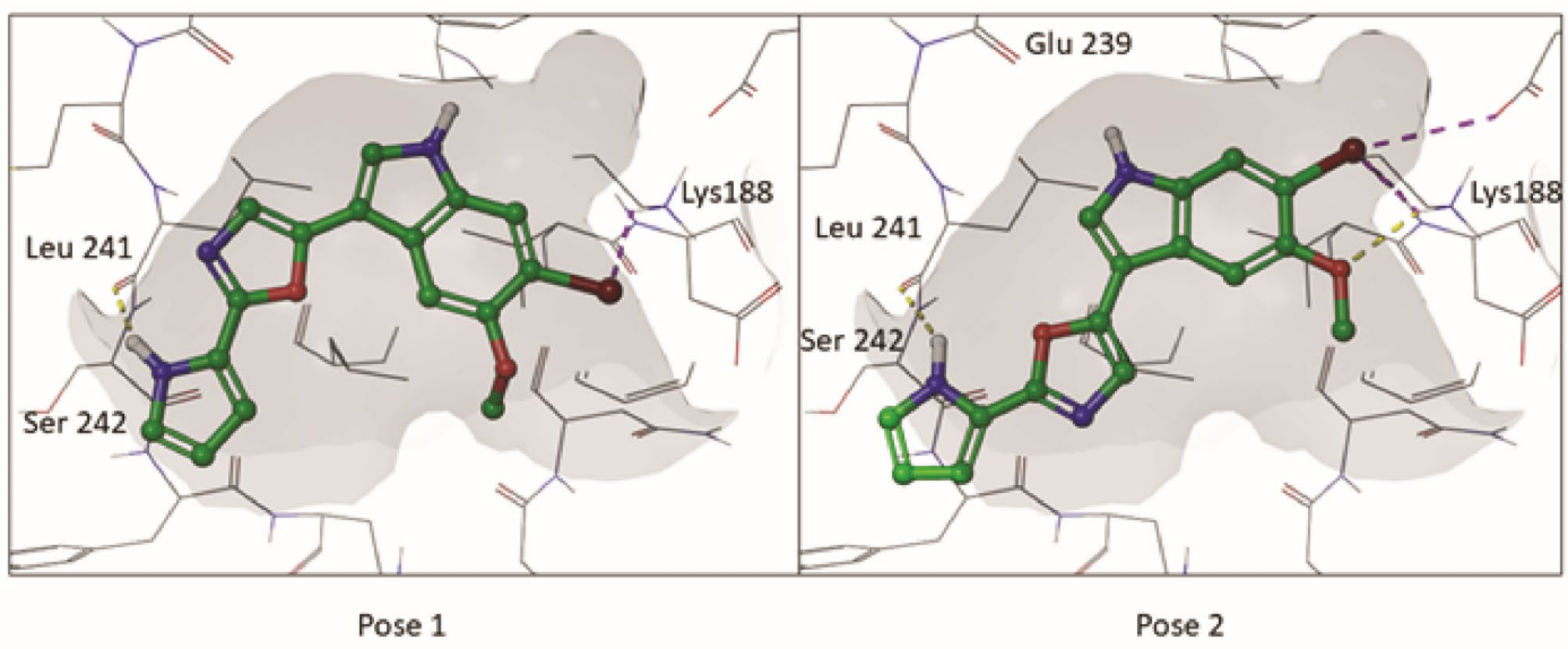
The two calculated poses of BfC (carbon atoms and bonds shown in green) in the ATP binding pocket of DYRK1A, PDB model 6S17. In both cases the pyrrole group of the compound forms a hydrogen bond with the kinase hinge (L241), while the indole seats in the hydrophobic pocket in a type 1 kinase inhibitor manner. The main difference between the two poses being the angles between the oxazole group and the rest of the molecule. Residue from the kinase is shown as grey wire, sulfur atoms are shown in yellow, oxygen in red, nitrogen in blue, bromine in maroon and polar hydrogens in white. Yellow dashed lines represent calculated hydrogen bonds and purple dashed lines represent halogen bonds. The binding pocket cavity is represented as a grey surface.

Docking simulations suggest two plausible poses for BfC in the ATP binding pocket of DYRK1A. In both cases BfC forms a hydrogen bond between the pyrrole group and the kinase hinge (L241), while the rest of the compound goes deeper into the hydrophobic pocket (Fig. 2) in a type 1 kinase inhibitor fashion. The main difference between the poses being the torsion angles of the oxazole group with the rest of the molecule and thus the position of the bromine and methoxy group in the back of the pocket. As control, the compounds harmine and 5IT were docked in the same DYRK1A model and adopted poses identical to that found in the DYRK1A crystal structure with PDB ID 3ANR for harmine, and very similar to that of crystal structure of CDK1 with PDB ID 6G33 for the compound 5IT (Supplementary Fig. 1, Supplementary Fig. 2).

### BfC inhibits the DYRK family of protein kinases

BfC inhibits the activity of the family of DYRK kinases with IC_50_ values of 1.3 ± 0.1 (DYRK1A), 1.4 ± 0.5 (DYRK2) and 4.4 ± 0.8 µM (DYRK3) (Table 1). Time-resolved fluorescence resonance energy transfer (TR-FRET) assay indicates binding to the kinase with a K_d_ of roughly 10 µM (DYRK1A) and roughly 9 µM (DYRK1B) (Table 1) (due to issues with compound autofluorescence, precise determination of the IC50 was obscured).

### BfC downregulates phosphorylation of DYRK1A/B and p27^KIP^

**Fig. 3:**
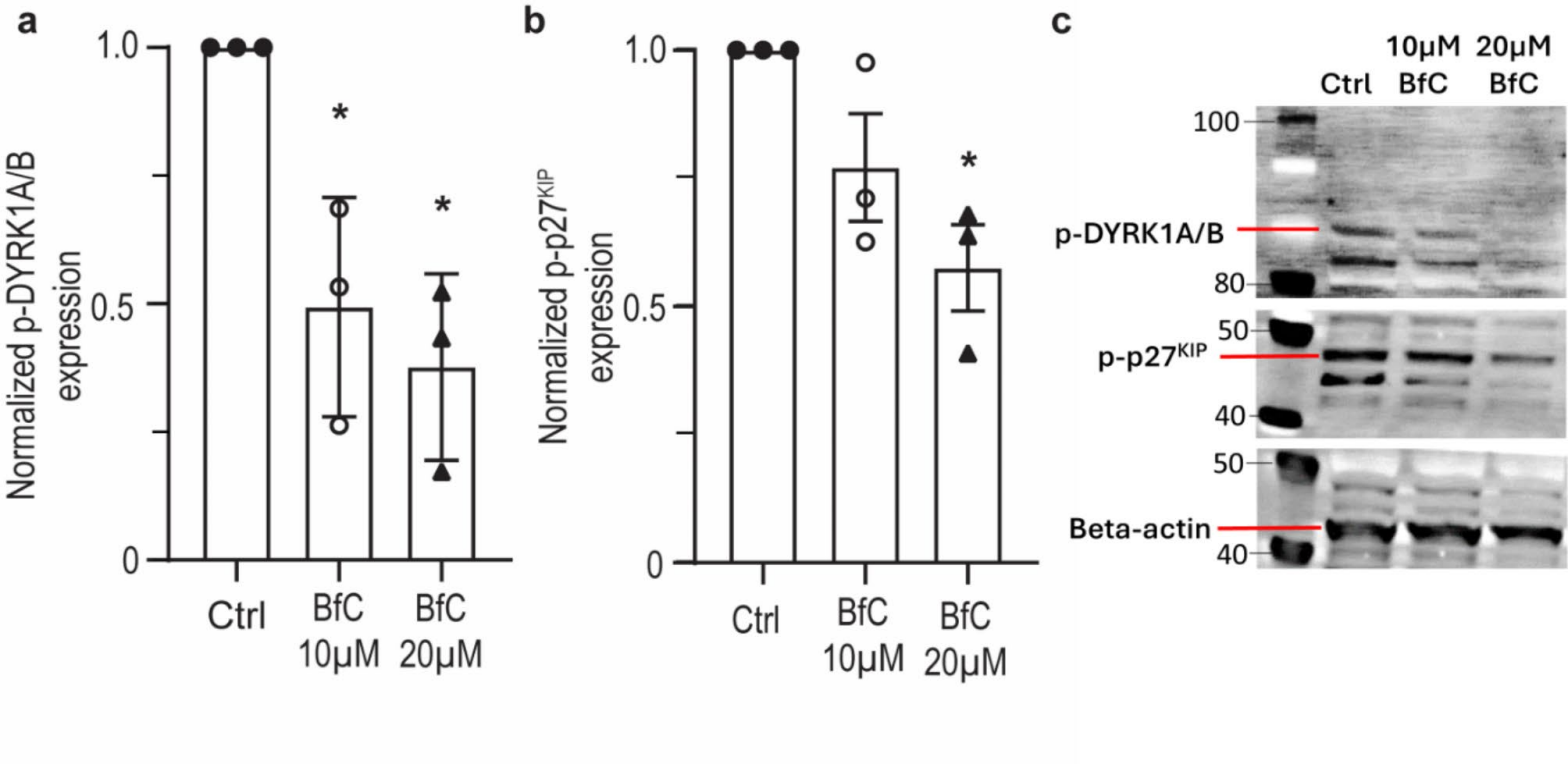
Western blot of phosphorylated DYRK1A/B (T321, T273) and p27^KIP^ (S10) in response to 48 h treatment with 10 µM and 20 µM BfC. (**A**) Treatment with 10 µM and 20 µM BfC significantly decreased the relative expression of phosphorylated DYRK1A/B compared to control. Data represent mean ± SEM of three individual experiments. *p < 0.05 compared to control, one-way ANOVA with Bonferroni’s post hoc test. (**B**) Treatment with 20 µM BfC significantly decreased the relative expression of phosphorylated p27^KIP^ compared to control. Data represent mean ± SEM of three individual experiments. *p < 0.05 compared to control, one-way ANOVA with Bonferroni’s post hoc test. (**C**): Representative western blot images for the datapoints shown in (**A**) and (**B**). Image of uncropped blots available in Supplementary Figure S4. Beta-actin was included as a loading control. Molecular ladder and reference weight in kDa, highlighted on the left. Closed circles = untreated control; open circles = cells treated with 10 µM BfC; closed triangles = cells treated with 20 µM BfC.

Treatment with 10 and 20 µM BfC (no cytokines) decreased the expression of p-DYRK1A/B by approximately 50% and 62% compared to control, respectively (Fig. 3, *n* = 3). Similarly, 10 and 20 µM BfC (no cytokines) decreased the expression of p-p27^KIP^ by 23% and 42% compared to control, respectively (Fig. 3, *n* = 3).

**Table 1:** Inhibition of DYRK kinases by breitfussin C analyzed by kinase activity and kinase binding assay.

Kinase activity assay	IC_50_ (µM)
DYRK1A	1.3 ± 0.1
DYRK2	1.4 ± 0.5
DYRK3	4.4 ± 0.8

Kinase binding assay	K_d_ (µM)
DYRK1A	10
DYRK1B	9

Data represents mean ± SD. Reference values for binding of 5IT to DYRK1A and B and literature IC_50_ values for 5IT inhibition are provided in the Supplementary Table S5.

## Discussion

This study reports the discovery of a new beta cell proliferative agent, BfC, isolated from the Arctic, marine invertebrate *Thuiaria breitfussi*. While members of the breitfussin family have been previously described as kinase inhibitors, the beta cell proliferative effect of BfC has not yet been documented.

Although most kinase inhibitors are known for their antineoplastic properties, their potential for other indications are also being evaluated. Emerging evidence from clinical and experimental data has suggested that off-target effects of TKIs may have beneficial impacts on diabetic patients^12^. It has been proposed that TKIs may not only reverse the progression of diabetes but also prevent its onset, as indicated by improvements in beta cell function and survival. The exact mechanisms by which kinase inhibitors exert beneficial effects on diabetic patients remain unclear, but theories suggest they may interfere with cytokine signalling^29,30^. Cytokines are critical in the progression of beta cell failure in diabetes, contributing to various adverse outcomes such as cellular dysfunction, increased apoptosis, and reduced proliferation and are considered promising targets for the treatment of diabetes and other inflammatory diseases^31,32^.

With the aim of exploring BfC’s potential in the diabetic therapeutic area, we investigated the ability of BfC to interfere with the pathophysiological signalling of pro-inflammatory cytokines. BfC treatment of RIN-M5F cells stimulated with IL-1β and IFN-γ significantly increased cell viability, suggesting a protective effect of BfC against these cytotoxic cytokines. Surprisingly, based on our results, BfC did not interfere with apoptosis, rather, it caused increased caspase–3/7 activity, a finding that may align with the compound’s previously reported polypharmacological profile^20^.

Given the anti-proliferative effects of cytokines^33^, we subsequently examined BfC’s mitogenic properties using EdU labelling. Remarkably, BfC not only increased EdU incorporation in untreated cells but also counteracted the cytokine-induced impairment of proliferation in cells stimulated by cytokines. To verify these findings, we performed cell cycle analysis. In line with our results, BfC treatment significantly increased the proportion of cells in the S- and G2/M phases, reinforcing the conclusion that treatment with BfC induces a proliferative state in the cells. However, it is still unclear whether BfC counteracts cytokines directly or whether it just increases cell proliferation.

The kinase inhibitory properties of BfC have previously been characterized, with initial profiling indicating that it inhibits several kinases, including DYRK1A^20^. DYRK1A has been recognized as a promising candidate for developing therapies that stimulate beta cell proliferation, suggesting a key mechanism through which BfC may promote the beta cell proliferative activity^13,14^. Through kinase inhibition assays, we confirmed BfC’s direct interaction with and inhibition of DYRK1A, DYRK1B, DYRK2, and DYRK3, with IC_50_ values ranging between 1 and 10 µM. Docking simulations supported these findings, suggesting that BfC can fit into the ATP-binding pocket of these kinases as a type 1 inhibitor.

To assess the inhibitory effects of BfC on cellular kinase signalling, we quantified the phosphorylation of DYRK1A/B and the downstream effector p27^KIP^. Western blot analysis revealed a significant decrease in phosphorylated DYRK1A/B and phosphorylated p27^KIP^ following BfC treatment. The cyclin-dependent kinase inhibitor p27^KIP^ is pivotal in cell cycle regulation, arresting cells in the G0/G1 phase, and its post-translational modifications are being essential for cell cycle progression^16^. Our results align with the hypothesis that DYRK1A activity impedes cell cycle progression by contributing to the stabilization of p27^34^. By inhibiting DYRK1A with BfC, a subsequent reduction in phosphorylated p27 allows cell cycle progression and mitotic entry, resulting in increased proliferation. This proposed mechanism is further supported by the effect of the well-known natural compounds 5-IT and harmine, which are documented to stimulate beta-cell proliferation through DYRK inhibition and are associated with a decreased phosphorylation of p27^13^. There is a possibility that that BfC boosts proliferation of beta-cells through additional mechanism unrelated to DYRK1A, but that has not been investigated in this manuscript.

Cell-specific proliferation is essential for ensuring oncological safety in the development of beta cell regenerative therapies. BfC was previously found to be cytotoxic to 48 cell lines at concentrations of 10 µM and below^20^, therefore we assume that the proliferative effect in beta cells investigated in this manuscript is at least partially specific. Previously, harmine in combination with a GLP-1 receptor agonist has indicated a significant induction of beta cell proliferation without off-target proliferative effects^14^. The proliferative selectivity of BfC needs to be further evaluated. In addition, our control experiments demonstrated that BfC treatment decreased EdU incorporation in HepG2 cells (Supplementary Fig. 3), indicating the possibility of a beta cell-specific mitogenic response to BfC. The current work is performed in the rodent beta cell line, RIN-M5F. Ideally, we hope to validate our findings in human islets as well as in vivo models once this scaffold has been refined to limit off target binding, as these models can be susceptible to adverse effect, making target deconvolution challenging and expensive, when looking at the effects of non-optimised natural products. Natural products frequently act as precursors or templates for subsequent drug development rather than directly as therapeutic agents. The characteristics of the unmodified natural product BfC reinforce its potential as a scaffold for a future beta cell proliferative therapy. Nonetheless, the ultimate success of BfC will depend on the future development process, which would aim to improve target potency and selectivity, improve pharmacokinetic and toxicological properties, and ensure patentability.

In conclusion, this study introduces BfC as a novel beta cell proliferative agent that functions as a small-molecule inhibitor of DYRK kinases, thereby expanding the family of naturally occurring DYRK inhibitors with potential to serve as a scaffold for drug development targeting beta cell proliferation.

## Electronic supplementary material

Below is the link to the electronic supplementary material.

Supplementary Material 1

## Data Availability

All the data presented is available in the manuscript and supplementary material files.
