# Supplementary material for: Promotion of beta cell proliferation through DYRK kinase inhibition using the marine natural product breitfussin C
Source: Sci Rep. 2025 Jan 8;15:1247. doi: 10.1038/s41598-025-85178-w (PMC11706957; doi:10.1038/s41598-025-85178-w)
Supplement: Supplementary file 1 — Supplementary Material 1 [file 41598_2025_85178_MOESM1_ESM.pdf]

Supplementary Material

Promotion of beta cell proliferation through DYRK kinase inhibition using the marine natural product breitfussin C

Sara Ullsten[1], Kine Østnes Hansen[1*], Guillaume Axel Petit[1], Espen Hansen[1], Jeanette Hammer Andersen[1*]

[1]MARBIO, UiT – The Arctic University of Norway, Breivika, N-9037, Tromsø, Norway

*** Correspondence:**
Jeanette Hammer Andersen

jeanette.andersen@uit.no

Supplementary Results

To assess the docking results with DYRK1A PDB model 6S17, compounds 5IT and harmine, were docked in this structure and compared to existing crystal structures of CLK1 with 5IT (PDB model 6G33, Fig S1) and DYRK1A with harmine (PDB model 3ANR, Fig S2). In both cases the computed poses and interaction with the kinase active site were almost identical to the crystal data, with some small differences for the solvent exposed part of the compounds in the case of 5IT.

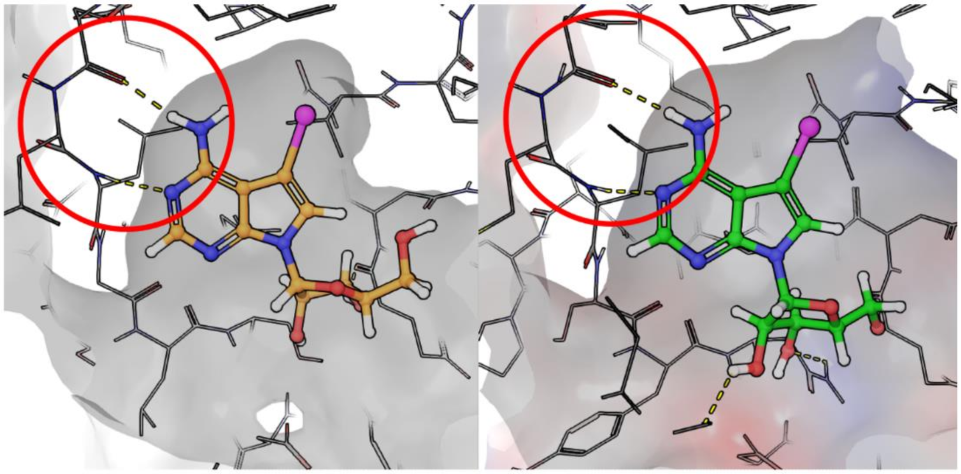

Crystal structure of CLK1 with 5IT (PDB ID 6G33)   Docking of 5IT in DYRK1A (PDB ID 6S17)

Supplementary Figure S1. Redocking of 5IT in DYRK1A. The interactions between the compounds and the kinase hinge are identical (red circles) in both calculated and experimental model, however the interactions with the solvent exposed regions are less conserved, which might be caused by the differences in the models used (CLK1 versus DYRK1A). These results suggests that the approach used is appropriate. The compounds are shown as cartoon, with blue for nitrogen atoms, white for hydrogen, red for oxygen, purple for iodine and either orange (experimental) or green (docked) for carbon atoms. The protein active site is shown as a grey surface and important residues are shown as wire, dashed line represent hydrogen bonds.

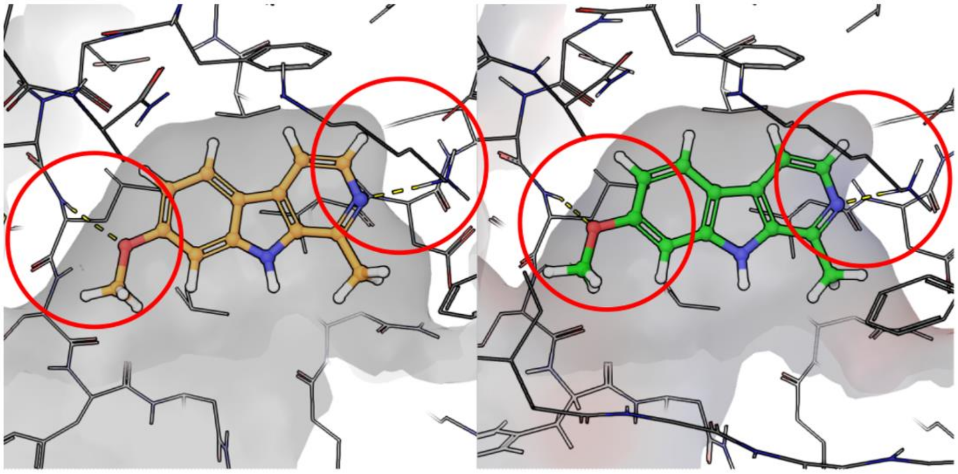

Crystal structure of DYRK1A with harmine (PDB ID 3ANR)   Docking of harmine in DYRK1A (PDB ID 6S17)

Supplementary Figure S2. Redocking of harmine in DYRK1A. The interactions between the compounds and the kinase hinge are identical (red circles) in both calculated and experimental model. Once again, these results are suggesting that the approach used in docking is appropriate. The compounds are shown as cartoon, with blue for nitrogen atoms, white for hydrogen, red for oxygen, purple for iodine and either orange (experimental) or green (docked) for carbon atoms. The protein active site is shown as a grey surface and important residues are shown as wire, dashed line represent hydrogen bonds.

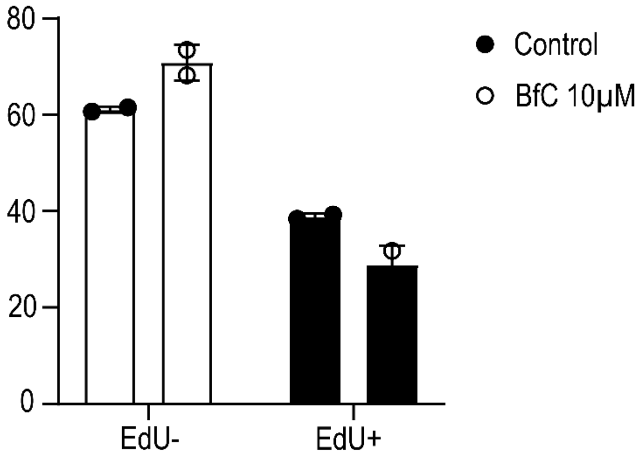

Supplementary Figure S3. Edu incorporation in HepG2 cells with and without BfC treatment (10 μM). Open bars: EdU negative cells. Closed bars: EdU positive cells. Closed circles: Untreated cells. Open circles: Cells treated with 10μM BfC.

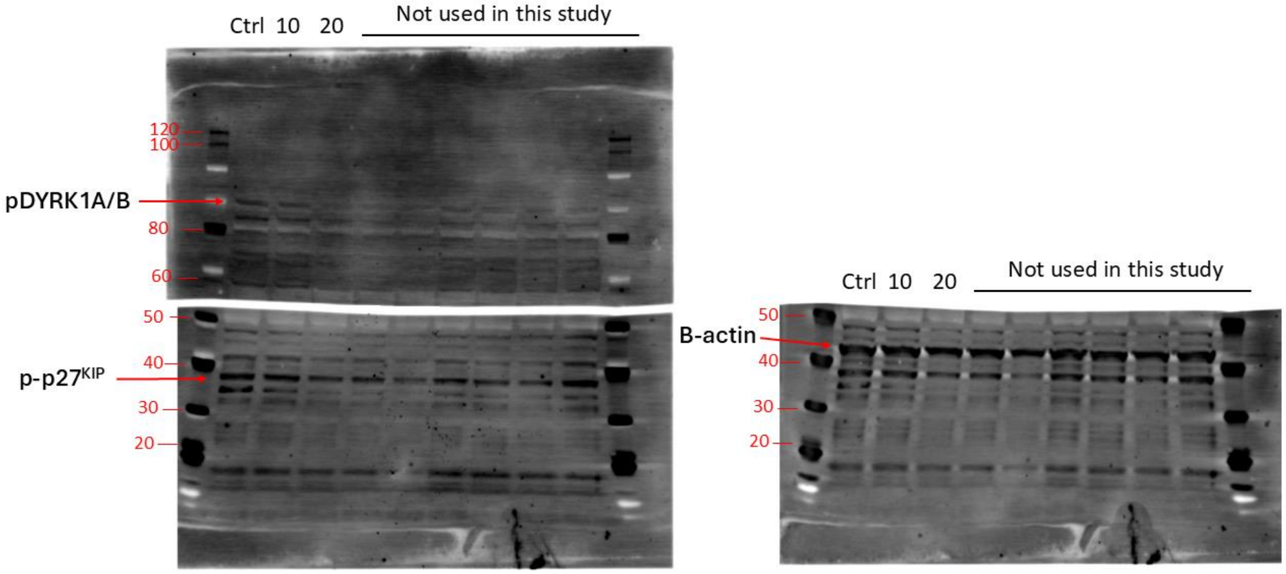

Supplementary Figure S4. Original western blot scan of the different targets. Molecular weights of the ladder are shown in red and given in kDa on the left hand side of each gel, the arrows indicate the band containing the target proteins. BfC treatment concentrations are indicated at the top of the three first lanes ─ no BfC (Ctrl), 10 µM BfC (10) and 20 µM BfC (20), the other lanes were not used as part of this study. The gels were cut in half before treatment with primary antibody. After imaging for p-P27^{KIP}, the bottom part was washed and re-stained for B-actin and imaged again.

Supplementary table S5. DYRK1A, and DYRK1B binding assay to 5IT.

**Binding assay**	**Kinase variant**	**K_d (µM)**
5IT	DYRK1A	0.0006 [0.0004; 0.0011]
5IT	DYRK1B	0.0024 [0.0017; 0.0030]
**Activity Assay**		**IC_{50} (µM)**
5IT	DYRK1A	0.013*
5IT	DYRK1B	0.010*

Binding of compounds to purified kinases using lanthaScreen technology, 95% confidence interval is indicated in bracket. *Reference activity values are taken from Dirice and colleagues (Dirice et al. 2016).

Supplementary references

Dirice, E., D. Walpita, A. Vetere, B. C. Meier, S. Kahraman, J. Hu, V. Dančík, S. M. Burns, T. J. Gilbert, D. E. Olson, P. A. Clemons, R. N. Kulkarni, and B. K. Wagner. 2016. 'Inhibition of DYRK1A Stimulates Human β-Cell Proliferation', *Diabetes*, 65: 1660-71.
